# Supplementary material for: Flexible utilization of spatial- and motor-based codes for the storage of visuo-spatial information
Source: eLife. 2022 May 6;11:e75688. doi: 10.7554/eLife.75688 (PMC9075954; doi:10.7554/eLife.75688)
Supplement: Transparent reporting form [file elife-75688-transrepform1.docx]

***eLife’s* transparent reporting form**

We encourage authors to provide detailed information *within their submission* to facilitate the interpretation and replication of experiments. Authors can upload supporting documentation to indicate the use of appropriate reporting guidelines for health-related research (see [EQUATOR Network](http://www.equator-network.org/%20)), life science research (see the [BioSharing Information Resource](https://biosharing.org/)), or the [ARRIVE guidelines](http://www.plosbiology.org/article/info:doi/10.1371/journal.pbio.1000412) for reporting work involving animal research. Where applicable, authors should refer to any relevant reporting standards documents in this form.

If you have any questions, please consult our Journal Policies and/or contact us: [editorial@elifesciences.org](mailto:editorial@elifesciences.org).

**Sample-size estimation**

- You should state whether an appropriate sample size was computed when the study was being designed
- You should state the statistical method of sample size computation and any required assumptions
- If no explicit power analysis was used, you should describe how you decided what sample (replicate) size (number) to use

Please outline where this information can be found within the submission (e.g., sections or figure legends), or explain why this information doesn’t apply to your submission:

The sample size (n=6) can be found in Materials and Methods – participants. To determine an appropriate sample size, we looked to other fMRI studies with similar methods to ours (e.g. Rademaker, Chunharas & Serences, 2019, Nature Neuroscience; Harrison & Tong, 2009, Nature). These past studies found that a sample size similar to ours was appropriate for a design with multiple sessions (3-4 sessions) per subject and analyses performed in a within-subject manner.

**Replicates**

- You should report how often each experiment was performed
- You should include a definition of biological versus technical replication
- The data obtained should be provided and sufficient information should be provided to indicate the number of independent biological and/or technical replicates
- If you encountered any outliers, you should describe how these were handled
- Criteria for exclusion/inclusion of data should be clearly stated
- High-throughput sequence data should be uploaded before submission, with a private link for reviewers provided (these are available from both GEO and ArrayExpress)

Please outline where this information can be found within the submission (e.g., sections or figure legends), or explain why this information doesn’t apply to your submission:

This can be found in Materials and Methods – participants. We performed both biological replication (repeating same experiment in multiple subjects) and technical replication (each subject was scanned multiple times with the same task). One pilot subject was excluded from the final analysis because this participant did not complete all the scans due to time constraints; otherwise all data were included. No outliers were encountered or excluded.

**Statistical reporting**

- Statistical analysis methods should be described and justified
- Raw data should be presented in figures whenever informative to do so (typically when N per group is less than 10)
- For each experiment, you should identify the statistical tests used, exact values of N, definitions of center, methods of multiple test correction, and dispersion and precision measures (e.g., mean, median, SD, SEM, confidence intervals; and, for the major substantive results, a measure of effect size (e.g., Pearson's r, Cohen's d)
- Report exact p-values wherever possible alongside the summary statistics and 95% confidence intervals. These should be reported for all key questions and not only when the p-value is less than 0.05.

Please outline where this information can be found within the submission (e.g., sections or figure legends), or explain why this information doesn’t apply to your submission:

Statistical methods can be found in Materials and Methods, in subsections Analysis: Univariate, Analysis: Spatial Position Decoding, and Analysis: Action Decoding. Statistical methods are also summarized in the legends of each figure. Raw data are presented on many of our figures (Figure 1C, 2B, 3A, 4), and elsewhere we display error bars that indicate +/- 1 SEM.

(For large datasets, or papers with a very large number of statistical tests, you may upload a single table file with tests, Ns, etc., with reference to sections in the manuscript.)

**Group allocation**

- Indicate how samples were allocated into experimental groups (in the case of clinical studies, please specify allocation to treatment method); if randomization was used, please also state if restricted randomization was applied
- Indicate if masking was used during group allocation, data collection and/or data analysis

Please outline where this information can be found within the submission (e.g., sections or figure legends), or explain why this information doesn’t apply to your submission:

We did not allocate subjects into groups, because we used a within-subject design where each subject participated in multiple conditions of the experiment. We used randomization to determine the order in which the conditions were presented to each subject (see Materials and Methods – Task: Main Working Memory). No masking of conditions was performed.

**Additional data files (“source data”)**

- We encourage you to upload relevant additional data files, such as numerical data that are represented as a graph in a figure, or as a summary table
- Where provided, these should be in the most useful format, and they can be uploaded as “Source data” files linked to a main figure or table
- Include model definition files including the full list of parameters used
- Include code used for data analysis (e.g., R, MatLab)
- Avoid stating that data files are “available upon request”

Please indicate the figures or tables for which source data files have been provided:

All data (i.e. the data for all figures and tables) is available publicly on Open Science Framework. All experimental and analysis code is available on Github (https://github.com/mmhenderson/wm_flex).
